# The relationship between physical activity and quality of life among community-dwelling middle-aged and older adults with type 2 diabetes: a chain mediation model of psychological resilience and exercise self-efficacy

**DOI:** 10.3389/fendo.2026.1784397

**Published:** 2026-05-19

**Authors:** Wanying Su, Jiang Xie, Hui Wu, Chao Yang, Xuan Dai, Lin Ma, Sirui Lu, Yan Chen

**Affiliations:** 1 Joint Surgery and Sport Medicine Department, Hunan Provincial People’s Hospital (The First Affiliated Hospital of Hunan Normal University), Changsha, Hunan, China; 2 Nursing Department, Hunan Normal University Hunan Provincial People’s Hospital, Changsha, China; 3 Nursing Department, Hunan Provincial People’s Hospital (The First Affiliated Hospital of Hunan Normal University), Changsha, Hunan, China; 4 Department of Burns and Wound Repair Surgery, Shenzhen People’s Hospital (The First Affiliated Hospital, Southern University of Science and Technology; The Second Clinical Medical College, Jinan University), Shenzhen, Guangdong, China; 5 Department of Endocrinology, Hunan Provincial People’s Hospital (The First Affiliated Hospital of Hunan Normal University), Changsha, Hunan, China; 6 Graduate Department, Hunan Academy of Chinese Medicine, Changsha, Hunan, China

**Keywords:** chain mediation model, exercise self-efficacy, physical activity, psychological resilience, quality of life, type 2 diabetes mellitus

## Abstract

**Background:**

Middle-aged and older adults with type 2 diabetes mellitus (T2DM) often experience insufficient physical activity (PA) and reduced quality of life (QOL). Understanding how psychosocial factors are associated with this relationship is important for developing effective nursing interventions. Therefore, this study aimed to investigate the mediating roles of psychological resilience and exercise self-efficacy in the association between PA and QOL among community-dwelling populations.

**Methods:**

A cross-sectional survey was conducted among 328 middle-aged and older adults (≥ 45 years old) with T2DM in Changsha, China. Data were collected using validated instruments, including the International Physical Activity Questionnaire (IPAQ-SF), Connor-Davidson Resilience Scale (CD-RISC), Exercise Self-Efficacy Scale, and the Medical Outcomes Study 36-Item Short Form Health Survey (SF-36). Structural equation modeling and 5,000-bootstrap resampling were used to test mediation effects.

**Results:**

Given the high collinearity observed between physical activity and exercise self-efficacy in the partial mediation model, a full mediation model was retained as the final model. PA was indirectly and positively associated with QOL primarily through exercise self-efficacy (standardized indirect effect = 0.195, 95% CI [0.103, 0.287]). The sequential pathway through psychological resilience and exercise self-efficacy was also significant (standardized indirect effect = 0.009, 95% CI [0.002, 0.017]), whereas psychological resilience alone was not a significant mediator. The final model demonstrated acceptable fit (χ²/df = 3.26, RMSEA = 0.083, CFI = 0.960, NFI = 0.944, IFI = 0.961, TLI = 0.920).

**Conclusions:**

This study tested a chain mediation model suggesting that the observed association between PA and QOL may be partly explained by a sequential psychosocial pathway involving psychological resilience and exercise self-efficacy. These findings highlight the potential value of integrating psychological empowerment and behavioral strategies in future interventions aimed at supporting better QOL among middle-aged and older adults with T2DM.

## Introduction

Type 2 diabetes mellitus (T2DM) is a chronic metabolic disorder characterized by insulin resistance and progressive β-cell dysfunction (1, 2). It imposes a significant global public health burden, with over 538 million middle-aged and older adults affected in 2023 and projections exceeding 780 million by 2045, of which more than 90% are T2DM cases (3, 4). In China, the number of people living with diabetes has surpassed 140 million, making it the country with the largest diabetic population worldwide, and over half of them are middle-aged and older adults (≥ 45 years old) (3). Beyond its metabolic consequences, T2DM substantially impairs quality of life (QOL) across physical, psychological, and social domains (5, 6). Enhancing QOL has therefore become a critical goal in the long-term management of T2DM, particularly for community-dwelling middle-aged and older adults who primarily self-manage their condition outside clinical settings.

Physical activity (PA) is widely recognized as a low-cost, accessible, and potentially beneficial non-pharmacological approach for supporting metabolic control and QOL in individuals with T2DM (7–9). Regular PA has been associated with better insulin sensitivity, fewer diabetes-related symptoms, and better physical and mental well-being (10–12). However, fewer than 30% of middle-aged and older adults with T2DM meet WHO-recommended PA guidelines, and adherence remains a major challenge in community populations (13, 14). These findings suggest that the impact of PA on QOL may be mediated by additional psychological factors rather than being a direct effect alone.

Psychological factors such as psychological resilience and exercise self-efficacy are increasingly recognized as key internal resources that influence chronic disease outcomes (15). Psychological resilience reflects an individual’s ability to adapt positively in the face of stress or illness, while exercise self-efficacy refers to confidence in maintaining regular physical activity (16, 17). Prior studies suggest that psychological resilience may be associated with higher self-efficacy by fostering adaptive beliefs and perceived control, forming a sequential pathway of behavioral motivation (18). However, few studies have empirically examined whether PA is associated with QOL through a chain mediation mechanism involving both psychological resilience and exercise self-efficacy—particularly among community-dwelling middle-aged and older adults with T2DM. The lack of such evidence limits our understanding of how psychological and behavioral processes interact to influence patient-centered outcomes.

**Figure 1:**
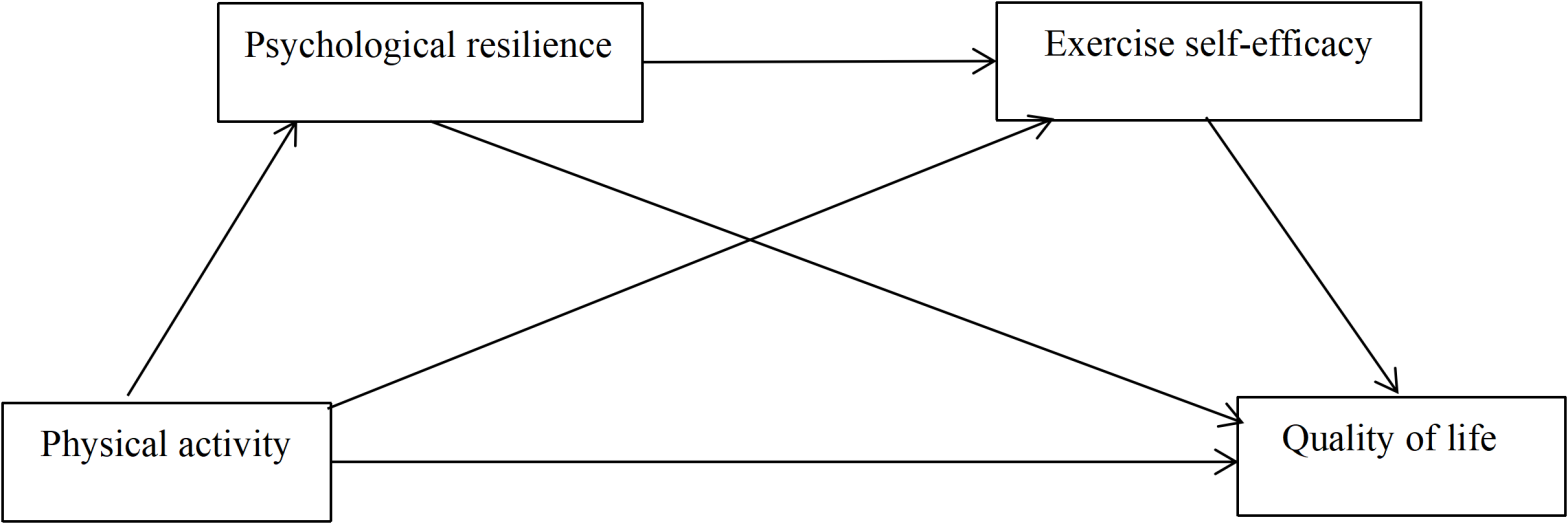
The hypothesized chain mediation effect model.

Therefore, this study adopts Rutter’s Developmental Model (19) as its theoretical framework, which posits that external protective factors (e.g., PA) may help foster internal protective resources (e.g., resilience and self-efficacy), thereby supporting adaptive functioning. Guided by this framework, the present study aimed to investigate the mediating roles of psychological resilience and exercise self-efficacy in the association between PA and QOL. To achieve this, we constructed and tested a structural equation model to examine four hypotheses: H1: PA is positively associated with QOL at the overall level; H2: psychological resilience mediates the PA–QOL relationship; H3: exercise self-efficacy mediates the PA–QOL relationship; H4: psychological resilience and exercise self-efficacy sequentially mediate the relationship between PA and QOL. By testing this model, we aim to elucidate the psychosocial mechanisms linking PA to QOL and provide actionable insights for developing targeted interventions in chronic disease management. The hypothesized model is shown in **Figure 1**.

## Methods

### Sample and data collection

This cross-sectional study adhered to the Strengthening the Reporting of Observational Studies in Epidemiology (STROBE) guidelines. Between December 1, 2024, and May 31, 2025, middle-aged and older adults with T2DM were recruited consecutively from four community health centers in Changsha, China, using a non-probability sampling approach. Inclusion criteria were: (1) diagnosis of T2DM based on the 1999 WHO criteria; (2) age ≥45 years; and (3) provision of written informed consent. Exclusion criteria included pregnancy or lactation, malignancy or severe complications, cognitive or psychiatric disorders, and physical impairments precluding PA. Ethical approval was granted by the institutional ethics committee (Approval No. 2024-217), and all procedures complied with the Declaration of Helsinki.

Sample size was determined *a priori* using G*Power software (version 3.1.9.7) (20). Because the core relationships in our final structural equation model can be approximated by multiple linear regression equations, an F-test for linear multiple regression (fixed model, R^2^ deviation from zero) was applied. Assuming a conservative small-to-medium effect size (f^2^ = 0.05), an alpha level of 0.05, a statistical power of 0.80, and 6 predictors in the largest regression equation, the minimum required sample size was estimated to be 279. A total of 347 middle-aged and older adults with T2DM were recruited, of whom 339 completed the survey. After excluding invalid or incomplete questionnaires, 328 valid responses were retained for analysis, which exceeded the minimum required sample size.

### Measures

#### Sociodemographic and clinical characteristics

Data on sociodemographic and clinical characteristics were collected using a structured questionnaire. Variables included age, gender, body mass index (BMI), educational level, marital status, living arrangement (e.g., with spouse, with children, or living alone), smoking and alcohol use, duration of diabetes, and comorbidities.

Physical activity (PA).

Physical activity was assessed using the short-form International Physical Activity Questionnaire (IPAQ-SF). The Chinese version of the IPAQ-SF has shown acceptable reliability and validity in Chinese adults in previous studies. The questionnaire comprises seven items, six of which assess physical activity at three intensity levels: walking (assigned a MET value of 3.3), moderate activity (MET = 4.0), and vigorous activity (MET = 8.0). Weekly energy expenditure was calculated using the formula (21):

#### MET-min/week = MET value × frequency (days/week) × duration (minutes/day)

Total physical activity was the sum of MET-min/week for all intensities and categorized as follows (22): (1) high-intensity PA (HI-PA): ≥3 days of vigorous activity totaling ≥1500 MET-min/week, or ≥7 days of any combination of activities totaling ≥3000 MET-min/week; (2) moderate-intensity PA (MI-PA): ≥3 days of vigorous activity for ≥20 min/day; or ≥5 days of moderate activity or walking for ≥30 min/day; or any combination achieving ≥600 MET-min/week; (3) low-intensity PA (LI-PA): does not meet the criteria for moderate or high levels.

#### Psychological resilience

Psychological resilience was assessed using the Chinese version of the Connor-Davidson Resilience Scale (CD-RISC). The original scale was developed by Connor et al. (23) to measure individuals’ resilience levels. It was later translated and culturally adapted into Chinese by Yu et al. (24). The scale consists of 25 items across three dimensions: tenacity, strength, and optimism. Each item is rated on a 5-point Likert scale ranging from 0 to 4, yielding a total score from 0 to 100. Higher scores indicate greater psychological resilience. The scale demonstrated good internal consistency, with a Cronbach’s alpha coefficient of 0.857.

#### Exercise self-efficacy

Exercise self-efficacy was assessed using the Exercise Self-Efficacy Scale, originally developed by Bandura (25) and later translated and adapted into Chinese by Tung et al. (26). The scale consists of 18 items, each rated on a scale from 0 (no confidence) to 100 (complete confidence). The final score is calculated as the average of all item scores, with higher values indicating greater exercise self-efficacy. The scale demonstrated excellent internal consistency, with a Cronbach’s alpha coefficient of 0.960.

#### Quality of life

Quality of life was measured using the Medical Outcomes Study 36-Item Short-Form Health Survey (SF-36). The Chinese version of the SF-36 has been widely translated, culturally adapted, and validated in mainland Chinese populations (27). It encompasses eight subscales across two domains: physical (physical functioning, role-physical, bodily pain, and general health) and mental (vitality, social functioning, role-emotional, and mental health) (28). Scores were transformed to a 0–100 scale using the following formula: Transformed Score = (Raw Score − Minimum Score)/Score Range × 100, with higher scores indicating better quality of life. The composite QOL score was the mean of the eight subscale scores. The scale demonstrated good internal consistency, with a Cronbach’s alpha coefficient of 0.819.

### Statistical analysis

Data analyses were performed using SPSS version 26.0 and AMOS version 24.0 (IBM Corp., Armonk, NY, USA). Continuous variables were expressed as mean (M) ± standard deviation (SD), while categorical variables were reported as frequencies and percentages. Differences in quality of life across sociodemographic characteristics and physical activity intensity levels were examined using independent-samples t-tests or one-way ANOVA. Descriptive statistics were used to summarize the distributions of physical activity, exercise self-efficacy, psychological resilience, and quality of life. Pearson correlation analysis was used to assess bivariate relationships among key variables.

Structural equation modeling (SEM) was employed to test the hypothesized path relationships among physical activity, psychological resilience, exercise self-efficacy, and quality of life. Prior to model specification, potential multicollinearity among predictors was assessed using bivariate correlations and variance inflation factors (VIFs). To address possible suppression-related distortion caused by collinearity and to retain a theoretically interpretable and parsimonious structure, a competing models strategy was used (29). Specifically, a partial mediation model was compared with a full mediation model, in which the direct path from physical activity to quality of life was constrained to zero. Age, gender, BMI, and diabetes duration were entered as covariates predicting quality of life to control for potential confounding. Model fit was evaluated using the Comparative Fit Index (CFI), Normed Fit Index (NFI), Incremental Fit Index (IFI), Tucker-Lewis Index (TLI), chi-square/degrees of freedom ratio (χ²/df), and Root Mean Square Error of Approximation (RMSEA). Acceptable model fit was defined as CFI, NFI, IFI, and TLI > 0.90, χ²/df < 5, and RMSEA < 0.10. Mediation effects were tested using bias-corrected bootstrap resampling with 5,000 samples, and statistical significance was determined by whether the 95% confidence intervals (CIs) excluded zero.

## Results

### Sample characteristics

**Table 1** presents the sociodemographic characteristics of the participants. A total of 328 individuals completed the questionnaire, including 157 males (47.9%) and 171 females (52.1%). In terms of age distribution, 135 participants (41.2%) were between 60 and 70 years old, another 135 (41.2%) were over 70 years, and 58 (17.6%) were younger than 60. Regarding marital status, the majority were married (n = 307, 93.6%). With respect to educational attainment, 68 participants (20.7%) had completed college or higher education, 77 (23.5%) had a high school or junior college education, 108 (32.9%) had a middle school education, and 75 (22.9%) had only primary education or no formal education.

**Table 1:** Sociodemographic characteristics of community-dwelling adults with type 2 diabetes (N = 328).

Variables	N (%)	QOL (M ± SD)	t/F	*P*-value
Age (years)			0.038	0.963
<60	58 (17.6)	74.58 ± 8.87		
60~70	135 (41.2)	74.23 ± 8.99		
>70	135 (41.2)	74.39 ± 9.19		
Gender			0.825	0.410
Male	157 (47.9)	74.83 ± 8.74		
Female	171 (52.1)	73.99 ± 9.58		
Body Mass Index (kg/m2)			2.548	0.080
<18.5	23 (7.0)	74.20 ± 8.34		
18.5-23.9	181 (55.2)	73.43 ± 9.14		
≥24	124 (37.8)	75.83 ± 9.28		
Education level			0.184	0.907
Primary school and below	75 (22.9)	74.37 ± 8.68		
Middle school	108 (32.9)	74.67 ± 9.11		
High school or junior college	77 (23.5)	73.75 ± 10.26		
College and above	68 (20.7)	74.71 ± 8.71		
Marital status			0.294	0.769
Married	307 (93.6)	74.96 ± 7.55		
Divorced or widowed	21 (6.4)	74.35 ± 9.30		
Living arrangement			0.712	0.545
With spouse	163 (49.7)	74.29 ± 9.34		
With children	41 (12.5)	72.73 ± 8.57		
With Spouse and children	90 (27.4)	73.24 ± 8.99		
Living alone	34 (10.4)	74.65 ± 9.79		
Working status			1.013	0.312
Yes	40 (12.2)	75.77 ± 8.33		
No	288 (87.8)	74.20 ± 9.30		
Smoking status			0.966	0.382
Never smoked	238 (72.6)	74.45 ± 9.42		
Currently smoke	38 (11.6)	72.67 ± 8.54		
Ever smoked	52 (15.9)	75.38 ± 8.54		
Alcohol consumption			0.591	0.554
Never drank	261 (79.6)	74.53 ± 9.44		
Currently drink	24 (7.3)	72.43 ± 8.14		
Ever drank	43 (13.1)	74.66 ± 8.15		
Duration of diabetes (year)			1.884	0.154
≤5	136 (41.5)	74.39 ± 8.99		
6~10	72 (22.0)	72.73 ± 9.07		
>10	120 (36.6)	75.39 ± 9.40		
Insulin therapy			-0.676	0.499
Yes	81 (24.7)	74.99 ± 8.79		
No	247 (75.3)	74.20 ± 9.33		
Oral antidiabetic drugs			0.864	0.388
Yes	279 (85.1)	74.21 ± 9.12		
No	49 (14.9)	75.44 ± 9.59		
Hypoglycemia			-0.425	0.671
Yes	86 (26.2)	74.03 ± 9.02		
No	242 (73.8)	74.52 ± 9.26		
Diabetic complications			0.574	0.566
Yes	71 (21.6)	74.95 ± 10.01		
No	257 (78.4)	74.24 ± 8.96		

QOL, quality of life; M, Mean; SD, standard deviation.

### Correlation between quality of life and sociodemographic and clinical characteristics

As shown in **Table 1**, the results of independent-samples t-tests and one-way ANOVA indicated no statistically significant associations between sociodemographic or clinical characteristics and quality of life at the bivariate level. Nevertheless, based on theoretical considerations and their potential confounding roles, age, gender, BMI, and diabetes duration were included as covariates in the subsequent SEM.

### Common method bias

To reduce potential common method bias (CMB), anonymous coding was employed during the data collection process to minimize bias introduced by self-reported measures. Additionally, Harman’s single-factor test was conducted using SPSS 26.0 to assess the presence of CMB. An exploratory factor analysis of all measurement items identified 10 factors with eigenvalues greater than 1. The first factor accounted for 15.71% of the total variance, which is well below the commonly accepted threshold of 40%, suggesting that CMB was not a significant issue in this study.

### Correlations between physical activity, exercise self-efficacy, psychological resilience, and quality of life

**Table 2** shows the correlation results among the key study variables. Physical activity was positively correlated with psychological resilience (*r* = 0.522, *p* < 0.001), exercise self-efficacy (*r* = 0.852, *p* < 0.001), and quality of life (*r* = 0.356, *p* < 0.001). Psychological resilience was positively associated with exercise self-efficacy (*r* = 0.542, *p* < 0.001), and quality of life was positively correlated with both exercise self-efficacy (*r* = 0.231, *p* < 0.001) and psychological resilience (*r* = 0.231, *p* < 0.001).

**Table 2:** Correlations between physical activity, exercise self-efficacy, psychological resilience, and quality of life (N = 328).

Variables	M	SD	1	2	3	4
1.physical activity	1479.64	494.64	–			
2.psychological resilience	68.40	15.38	0.522^**^	–		
3.exercise self-Efficacy	60.26	24.79	0.852^**^	0.542^**^	–	
4.quality of Life	74.52	9.18	0.356^**^	0.231^**^	0.231^**^	–

M, Mean; SD, standard deviation; **P<0.001.

Association Between Physical Activity Intensity and Quality of Life Among Community-Dwelling Middle-Aged and Older Adults With Type 2 Diabetes **Table 3** presents differences in quality-of-life scores across physical activity intensity groups among community-dwelling middle-aged and older adults with type 2 diabetes. Significant between-group differences were observed across intensity strata (*p* < 0.05), suggesting a graded association between physical activity intensity and quality of life.

**Table 3:** One-way ANOVA results for the effects of physical activity on quality of life among community-dwelling middle-aged and older adults with type 2 diabetes (N = 328).

Variables	Exercise amount (M ± SD)			F	P
	HI-PA (n= 38)	MI-PA (n= 183)	LI-PA (n= 107)		
The quality of life	76.63 ± 9.72	76.21 ± 8.75	70.49 ± 8.55	15.648	0.000^**^

HI-PA, highintensity PA; MI-PA, moderate-intensity PA; LI-PA, low-intensity PA; M,Mean; SD,standard deviation (SD); **P<0.001.

### Analysis of mediation effects

To determine the final structural model, a competing-models strategy was adopted. First, a partial mediation model, including the direct path from physical activity to quality of life, was tested. Physical activity and exercise self-efficacy were highly correlated (*r* = 0.852), and their VIFs were 3.58 and 3.75, respectively, suggesting notable collinearity between the two variables and possible instability when the direct and mediated paths were estimated simultaneously. Given that the primary aim of this study was to examine the mediating roles of psychological resilience and exercise self-efficacy in the association between physical activity and quality of life, a full mediation model was further tested by constraining the direct path from physical activity to quality of life to zero, while simultaneously retaining age, gender, BMI, and diabetes duration as covariates predicting quality of life. The results showed that the full mediation model provided a more stable and theoretically interpretable representation of the relationships among the study variables and was therefore retained as the final model. The final model demonstrated acceptable fit to the data, with χ²/df = 3.26, RMSEA = 0.083, CFI = 0.960, NFI = 0.944, IFI = 0.961, and TLI = 0.920.

**Figure 2:**
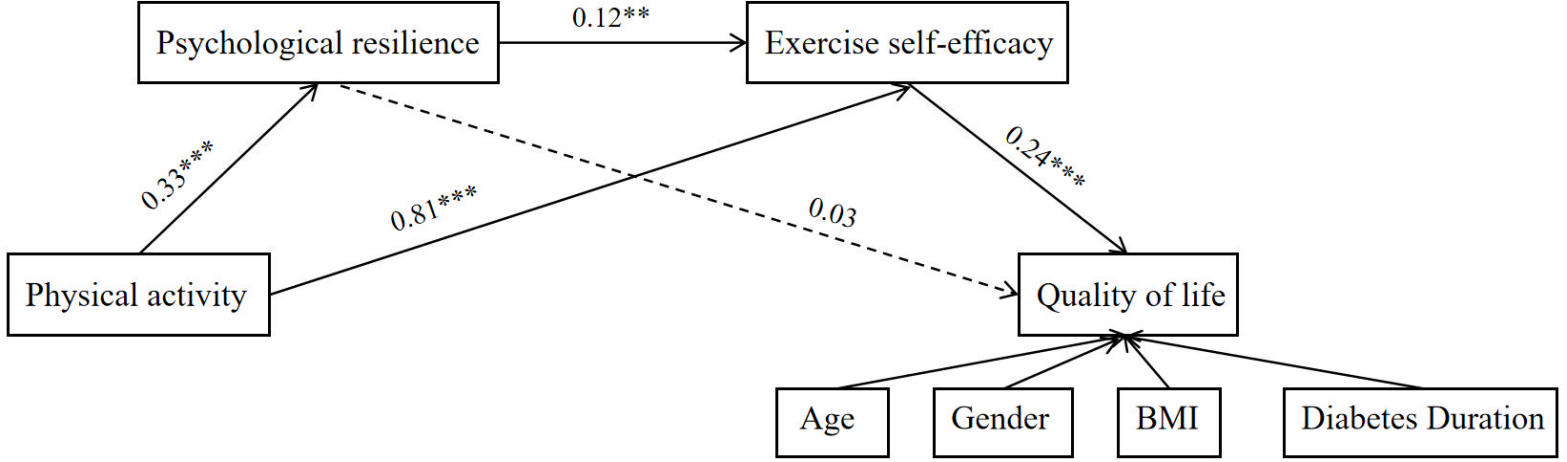
A chain mediation model of psychological resilience and exercise self-efficacy in the association between physical activity and quality of life. *p<0.05, **p<0.01, ***p<0.001. The non-significant path is indicated by a dashed line.Covariates including age, gender, BMI, and duration of diabetes were controlled in the model predicting quality of life.

In addition, **Figure 2** illustrates the standardized path coefficients of the final full mediation model. Because a full mediation model was adopted, the direct path from physical activity to quality of life was constrained to zero; therefore, the hypothesized direct effect was not retained in the final model. Physical activity significantly predicted psychological resilience (*β* = 0.327, *p* < 0.001) and exercise self-efficacy (*β* = 0.811, *p* < 0.001). Psychological resilience also significantly predicted exercise self-efficacy (*β* = 0.118, *p* = 0.001). In turn, exercise self-efficacy significantly and positively predicted quality of life (*β* = 0.241, *p* < 0.001), whereas the path from psychological resilience to quality of life was not statistically significant (*β* = 0.032, *p* > 0.05). These findings indicate that the indirect pathway physical activity → exercise self-efficacy → quality of life was supported, whereas the indirect pathway physical activity → psychological resilience → quality of life was not supported. In addition, the sequential pathway physical activity → psychological resilience → exercise self-efficacy → quality of life was also supported.

**Table 4** summarizes the total effect and specific indirect effects estimated from the final full mediation model. A bias-corrected bootstrap procedure with 5,000 resamples was used to assess the significance of the mediation effects. The total effect of physical activity on quality of life was significant (effect = 0.215, 95% CI [0.130, 0.300], *p* < 0.001). Among the indirect effects, the pathway physical activity → exercise self-efficacy → quality of life was statistically significant (effect = 0.195, 95% CI [0.103, 0.287], *p* < 0.001), and the sequential pathway physical activity → psychological resilience → exercise self-efficacy → quality of life was also statistically significant (effect = 0.009, 95% CI [0.002, 0.017], *p* = 0.018). By contrast, the pathway physical activity → psychological resilience → quality of life was not statistically significant (effect = 0.010, 95% CI [−0.026, 0.047], *p* = 0.572). Because the direct path was constrained to zero in the final full mediation model, the total effect was entirely composed of indirect effects.

**Table 4:** Total and indirect effects of physical activity on quality of life in the final full mediation model (N = 328).

Path	Estimate	Boot SE	95 CI	P
Total Indirect effect	0.215	0.043	[0.130, 0.300]	<0.001
Indirect effect (Physical activity →Psychological resilience → Quality of life)	0.010	0.019	[-0.026, 0.047]	0.572
Indirect effect (Physical activity →Exercise self-efficacy → Quality of life)	0.195	0.047	[0.103, 0.287]	<0.001
Indirect effect (Physical activity →Psychological resilience → Exercise self-efficacy → Quality of life)	0.009	0.004	[0.002, 0.017]	0.018

Data are reported as standardized coefficients; the Bootstrap sample size is set at 5000; SE, standard error; CI, confidence interval. In the final full mediation model, the direct path from physical activity to quality of life was constrained to zero; therefore, the total effect was entirely composed of indirect effects. Age, gender, BMI, and diabetes duration were controlled as covariates in the model.

## Discussion

Guided by Rutter’s Developmental Model, our study systematically investigated the relationships among PA, psychological resilience, exercise self-efficacy, and QOL in community-dwelling middle-aged and older adults with T2DM. Explicitly mapping our variables to Rutter’s framework, PA was conceptualized as an external protective behavior, psychological resilience as a foundational internal trait, and exercise self-efficacy as a specific cognitive adaptation. Our findings suggest that PA was associated with QOL at the bivariate level and that this association may be partly explained by psychosocial pathways involving psychological resilience and exercise self-efficacy. It is also important to note that the final model retained in this study was selected based on both statistical stability and theoretical interpretability. Because physical activity and exercise self-efficacy were highly correlated, the simultaneous estimation of direct and indirect paths in the partial mediation model produced unstable coefficients, suggesting potential coefficient instability and possible suppression-like effects. The full mediation model therefore provided a more stable and theoretically coherent representation of the relationships among the study variables. These findings offer insight into the psychosocial mechanisms that may help explain variations in QOL in this population.

First, our bivariate analysis showed a significant positive association between PA and QOL, consistent with previous findings (30, 31), and supporting the well-established role of PA as a safe and cost-effective non-pharmacological strategy for individuals with chronic conditions (32). Previous research suggests that regular PA is associated with better glycemic control, fewer diabetes-related symptoms, and improved overall well-being (33). In our sample, higher levels of PA were associated with higher QOL scores. This pattern is also consistent with prior evidence suggesting that moderate-to-high intensity PA may offer greater health benefits by supporting metabolic function, reducing fatigue, promoting social interaction, and improving sleep quality (34, 35). However, although these broad physiological and behavioral benefits are well documented, our final structural model suggested that the observed association between PA and QOL in this sample may be partly conveyed through psychosocial pathways. In addition, 32.6% of participants in this study reported low levels of PA, possibly because of comorbidities, limited physical capacity, or concerns about injury or hypoglycemia. Hence, future interventions should adopt individualized approaches and consider incorporating low-intensity, high-adherence activities (e.g., walking and Baduanjin (36)), given their feasibility and their potential to support better QOL in this population.

In contrast, psychological resilience alone did not emerge as a significant independent mediator between PA and QOL. Although psychological resilience is widely recognized as a protective factor in chronic disease contexts (37), its direct association with QOL was not statistically significant in this study. One possible explanation is that psychological resilience may function as a broader, upstream psychological resource rather than as a direct determinant of perceived quality of life (38). In other words, resilience may help individuals adapt to illness-related stress, but its influence on QOL may depend on whether this general adaptive capacity is translated into more proximal and behavior-specific cognitive resources. This interpretation is also consistent with our final model, in which psychological resilience significantly predicted exercise self-efficacy but did not directly predict QOL. Another possible explanation is that participants in this community-based sample may have been exposed to ongoing contextual stressors, such as financial strain, multiple comorbidities, or limited social support, which may have weakened the direct contribution of resilience to QOL (39). Taken together, these findings suggest that psychological resilience may still play an important role in the psychosocial pathway linking PA to QOL, but that its effect is more likely to operate indirectly through more proximal mechanisms such as exercise self-efficacy.

Moreover, our study supported the mediating role of exercise self-efficacy in the association between PA and QOL. Exercise self-efficacy, as a central construct in Bandura’s Social Cognitive Theory, reflects a more proximal, behavior-specific belief about one’s confidence in performing and sustaining physical activity, and it may therefore play an important role in shaping behavioral engagement and persistence (40). Our results suggest that greater engagement in PA may be associated with higher exercise self-efficacy, allowing individuals to experience positive feedback and develop a reinforcing loop of behavior, cognition, and outcome. Exercise self-efficacy may also help buffer the effects of physical and psychological challenges, thereby supporting sustained health behavior (41). The exercise self-efficacy findings highlight the potential of exercise self-efficacy as a modifiable psychological resource and underscore the value of incorporating exercise self-efficacy-enhancing strategies into behavioral interventions targeting T2DM populations.

Most importantly, the sequential mediation analysis suggested that the association between PA and QOL may be partly explained by the chain pathway of psychological resilience and exercise self-efficacy, consistent with the theoretical pathway of “psychological regulation → behavioral motivation → health outcome” widely proposed in behavioral health models (18). As a foundational emotional and cognitive resource, psychological resilience may not exert a direct impact on quality of life but rather initiates a chain of psychological adaptations that enhance individuals’ capacity to engage in health-promoting behaviors. Specifically, individuals with greater resilience may be more likely to reinterpret illness-related barriers—such as physical discomfort, fatigue, or lack of social support—as manageable challenges, thereby strengthening their confidence to maintain regular physical activity, i.e., exercise self-efficacy (42, 43). Once established, this task-specific confidence may reinforce continued engagement in physical activity, fostering a positive feedback loop of psychological reinforcement and behavioral engagement. This, in turn, may be associated with higher QOL (44). The chain mediation model thus provides a more nuanced understanding of how broader emotional resources may be translated into perceived well-being through more proximal behavioral mechanisms. From a practical perspective, these findings suggest that interventions aimed at improving QOL among individuals with T2DM should not focus solely on strengthening psychological resilience. Instead, they may benefit from an integrated approach that simultaneously builds exercise self-efficacy and facilitates behavior change.

### Limitations and future directions

Despite the novel insights and practical implications of our study, several limitations should be acknowledged: (1) The study employed a cross-sectional design, which precludes causal inferences regarding the relationships among variables. Although our model specified a directional pathway, bidirectional relationships may exist. For example, individuals with higher QOL may be more likely to engage in PA, while psychological resources and health behaviors may also influence each other over time. Future research should utilize longitudinal or experimental designs to clarify temporal ordering and causal mechanisms underlying the chain mediation model; (2) The sample was recruited from four community health centers in Changsha, Hunan Province, which may limit the generalizability of the findings. Future studies should include more diverse populations from different geographic regions, urban and rural settings, and varied socioeconomic backgrounds to improve the external validity of the results; (3) Data collection relied primarily on self-reported questionnaires, which may be subject to social desirability and recall biases. In particular, PA was measured using the IPAQ-SF, which is known to have potential overestimation bias in older or clinical populations. This reliance on self-report may have inflated the observed associations. In addition, the high correlation between PA and exercise self-efficacy suggests a degree of shared variance between behavioral performance and behavior-specific confidence in this population. Moreover, the model included only two mediating variables—psychological resilience and exercise self-efficacy —excluding other potentially relevant psychosocial constructs such as emotion regulation, health beliefs, and social support. Future research is encouraged to integrate objective measurements (e.g., accelerometers for PA) and more comprehensive theoretical models to enhance data accuracy and expand the explanatory framework.

## Conclusion

This study developed and tested a structural model illustrating how physical activity is associated with quality of life among community-dwelling middle-aged and older adults with T2DM. The findings suggest that the observed association between physical activity and quality of life may be partly explained by a sequential psychosocial pathway, in which physical activity is linked to enhanced general psychological resilience, which in turn supports specific exercise self-efficacy. These findings highlight the potential value of integrated interventions that simultaneously target behavioral activation and psychological empowerment to support better quality of life in middle-aged and older adults with T2DM.

## Data Availability

The raw data supporting the conclusions of this article will be made available by the authors, without undue reservation.
